# Supplementary material for: Vitamin D Modified DSS-Induced Colitis in Mice via STING Signaling Pathway
Source: Biology (Basel). 2025 Jun 18;14(6):715. doi: 10.3390/biology14060715 (PMC12190092; doi:10.3390/biology14060715)
Supplement: Supplementary file 1 [file biology-14-00715-s001.zip › Table S1: method of 16s-gut microbiota analysis.pdf]

MATERIALS AND METHODS

Sample collection and satorage

As we known, different sample collection, storage methods even proposed sampling site will have an impact on the results of 16s sequencing of intestinal flora. Though there is conflicting evidence on whether different storage conditions alone can have an impact on microbial community studies. It is often not practical to extract DNA from fresh samples; therefore, samples are generally stored for various flora stabilizing agent prior to DNA extraction.
Consequently, when considering the use of a cryoprotectant for storage, it is important to ensure that all samples are stored in the same manner. The present study explored the effects of using GUHE Flora Storage buffer (Zhejiang Hangzhou Equipment Preparation 20190682, GUHE Laboratories, Hangzhou, China).

DNA Extraction

Total bacterial genomic DNA samples were extracted using the Metagenomic DNA was extracted from all Samples using the GHFDE100 (Zhejiang Hangzhou Equipment Preparation 20190952) DNA isolation kit (GUHE Laboratories, Hangzhou, China), following the manufacturer ’s instructions. Incidentally, This extraction method has also obtained a Chinese national invention patent (ZL201511009389.7). The quantity and quality of extracted DNAs were measured using a NanoDrop ND-1000 spectrophotometer (Thermo Fisher Scientific, Waltham, MA,USA) and agarose gel electrophoresis, respectively.

16S rDNA Amplicon Pyrosequencing

PCR amplification of the bacterial 16S rRNA genes V4 region was performed using the forward primer 515F (5’- GTGCCAGCMGCCGCGGTAA -3’) and the reverse primer 806R (5’-GGACTACHVGGGTWTCTAAT-3’). Sample-specific paired-end 6-bp barcodes were incorporated into the TrueSeq adaptors for multiplex sequencing. The PCR components contained 25 μl of Phusion High-Fidelity PCR Master Mix, 3 μl (10 uM) of each Forward and Reverse primer, 10 μl of DNA Template, 3μl of DMSO, and 6 μl of ddH2O. Thermal cycling consisted of initial denaturation at 98 °C for 30 s, followed by 25 cycles consisting of denaturation at 98 °C for 15 s, annealing at 58 °C for 15 s, and extension at 72 °C for 15 s, with a final extension of 1 min at 72 °C. PCR amplicons were purified with Agencourt AMPure XP Beads (Beckman Coulter, Indianapolis, IN) and quantified using the PicoGreen dsDNA Assay Kit (Invitrogen, Carlsbad, CA, USA). After the individual quantification step, amplicons were pooled in equal amounts, and pair-end 2×150 bp sequencing was performed using the Illlumina NovaSeq6000 platform at GUHE Info technology Co., Ltd (Hangzhou,China).

Sequence Analysis

Raw sequencing reads with exact matches to the barcodes were assigned to respective samples and identified as valid sequences. Paired-end reads were assembled using Vsearch V2.22.1(--fastq_mergepairs --fastq_minovlen 5).The Amplicon Sequence Variant (ASV) picking using Vsearch v2.22.1, included dereplication (--derep_fulllength), Perform quality control and denoising sequences with UNOISE2 algorithm (--cluster_unoise) (Edgar, 2016) , Chimera removal (uchime3_denovo) (Rognes 2016) and mapping to ASVs with 100% similarity threshold (--usearch_global).

To minimize the difference of sequencing depth across samples, normalization was performed on the ASV sequence counts of samples in the ASV Table, in order to ensure that the sum of ASV sequences in each sample was consistent. Normalized values were set to 1, representing relative abundances. A representative sequence (rep-seqs) was selected from each ASV using default parameters. Importing rep-seqs and ASV table files into QIIME2(QIIME2-2022.2).ASVs containing less than 0.001% of total sequences across all samples were discarded by QIIME2. Taxonomic identifications represented the respective sequences assigned to ASVs against the QIIME2 weighted taxonomic classifiers (silva-138-99-nb-weighted-classifier). The taxonomy generated was collapsed (Levels 1–7) using the "qiime taxa collapse" command.

Bioinformatics and Statistical Analysis

Alpha and beta diversity calculations were generated using the q2-diversity core-metrics phylogenetic tool from a ASV table. Alpha-diversity metrics included Chao1 richness estimator, ACE metric (Abundance-based Coverage Estimator), PD_whole_tree, Shannon diversity index and Simpson index, with statistically significant differences between groups identified using the Kruskal–Wallis test. For beta diversity, weighted UniFrac distances, unweighted UniFrac distances and Bray-Curtis distance was used for ordination via principal coordinates analysis (PCoA), with statistically significant differences between groups identified using analysis of similarity (ANOSIM) and multivariate variance analysis (Adonis) also called Permutational multivariate analysis of variance (PERMANOVA) using R package "vegan". Beta diversity analysis visualized via principal coordinate analysis (PCoA), principal component analysis (PCA) and nonmetric multidimensional scaling (NMDS).

Sequence data analyses were mainly performed using QIIME2 and R packages (v3.6.3). Venn diagram was generated to visualize the shared and unique ASVs among samples or groups using R package "VennDiagram", based on the occurrence of ASVs across samples/groups regardless of their relative abundance. Principal component analysis (PCA) was also conducted based on the ASV-level compositional profiles. The envfit function in the VEGAN R package was used to perform a permutation test to test for effects of environmental factors on community structure.

Taxa abundances at the phylum, class, order, family, genus and species levels were statistically compared among groups by Kruskal–Wallis rank-sum test (groups = 2) or Wilcoxon rank-sum test(groups > 2) from R stats package.Taxa abundances were tested for between-group differences using the Kruskal-Wallis test or Tukey test, and visualized through the box-and-whiskers plots. And taxa abundances was compared with the sample groups in pairs with the MetagenomeSeq method (R metagenomeSeq package). All statistical tests $P < 0.05$ was considered statistically significant.

LEfSe (Linear discriminant analysis effect size) was performed to detect differentially abundant taxa across groups using the default parameters. Random forest analysis was applied to discriminating the samples from different groups using the R package "randomForest" with 1,000 trees and all default settings. The generalization error was estimated using 10-fold cross-validation. The expected "baseline" error was also included, which was obtained by a classifier that simply predicts the most common category label. Analysis of receiver operation characteristics (ROCs) and areas under the curve (AUCs) were used to assess the accuracy of the diagnostic model. Function prediction analysis (KEGG enzymes and MetaCyc pathway) was performed using PICRUSt2. Similarly, FAPROTAX and BugBase was used to predict function from taxonomic profiles.The output file was further analysed using Statistical Analysis of Metagenomic Profiles (STAMP) software package v2.1.3.
